# Nursing Practice Environments and Professional and Care-Related Outcomes in Portuguese Emergency Services: A Descriptive Study of 2018 and 2022

**DOI:** 10.3390/nursrep16040111

**Published:** 2026-03-28

**Authors:** Ângela Pragosa, Sofia Roque, Beatriz Araújo, Élvio Jesus

**Affiliations:** 1 Center for Interdisciplinary Research in Health (CIIS), Faculdade de Ciências da Saúde e Enfermagem, Universidade Católica Portuguesa, 4169-005 Porto, Portugal; baraujo@ucp.pt (B.A.); ejesus@ucp.pt (É.J.); 2 School of Health Sciences, Polytechnic University of Leiria, 2411-901 Leiria, Portugal; 3 Center for Innovative Care and Health Technology (ciTechCare), Polytechnic of Leiria, 2414-016 Leiria, Portugal; 4 CARE—Research Center on Health and Social Sciences, Portalegre Politechnic University, 7300-555 Portalegre, Portugal

**Keywords:** nursing practice environment, patient safety, quality of health care, job satisfaction, intention to leave, missed nursing care, emergency service, hospital

## Abstract

**Background/Objectives**: Emergency Services (ESs) are highly demanding clinical settings where Nursing Practice Environments (NPEs) play a critical role in shaping professional- and care-related outcomes. International evidence suggests that unfavorable NPEs are associated with reduced job satisfaction, compromised care quality, and increased safety risks. This study aimed to describe NPEs and selected professional and care-related outcomes among ESs nurses in Portugal in 2018 and 2022. **Methods**: A descriptive, cross-sectional study was conducted using data from two national surveys of ESs nurses collected in 2018 (*n* = 390) and 2022 (*n* = 434). Data were collected through an online questionnaire including the Practice Environment Scale of the Nursing Work Index (PES-NWI), measures of job satisfaction, intention to leave, perceived quality and safety of care, safety culture, incident occurrence, and missed nursing care. Descriptive statistics were used to summarize results across both samples. **Results**: NPEs were predominantly classified as unfavorable in both samples, with around 70% of nurses working in unfavorable environments. The most compromised dimensions were staffing and resource adequacy, nurses’ participation in hospital affairs, and nurse manager ability, leadership, and support of nurses. Job satisfaction was low in both samples, and a high proportion of nurses reported an intention to leave the organization. Differences were observed between samples in perceived quality and safety of care, incident occurrence, and missed nursing care, particularly in relational and autonomous interventions. Collegial nurse–physician relations emerged as the only favorable dimension in both samples. **Conclusions**: The findings indicate that NPEs in Portuguese ESs were predominantly unfavorable in both study periods, reflecting structural and organizational challenges. These findings may be associated with nurses’ professional outcomes and perceived care quality and safety, highlighting the importance of targeted organizational interventions to improve practice environments.

## 1. Introduction

Concern about the study of practice environments is not recent. As early as the 19th century, Florence Nightingale examined care environments in field hospitals and implemented improvements in hygiene and nutrition to enhance patient safety, observing a substantial reduction in deaths and care-associated infections [1].

Concerns about NPEs arose in the United States during the 1970s amid hospital nurse shortages. Despite this situation, some institutions consistently succeeded in attracting and retaining nurses. These organizations, later designated as Magnet Hospitals, drew scientific interest for their high retention rates. To understand this phenomenon, the organizational traits of these hospitals were studied using nurses’ firsthand accounts of why they stayed at their workplaces. The analysis identified common features across 41 Magnet hospitals, including decentralized decision-making at the unit level, strong, effective, and visible nursing leadership, recognition of nurses’ professional autonomy, accountability for the quality of care, adequate staffing, and more flexibility in scheduling [2,3].

NPEs currently assume a central, conceptually well-established role in research on healthcare quality and safety, constituting one of the most robust frameworks for understanding the relationships among organizational contexts, care processes, and clinical and professional outcomes [2,4,5,6]. Defined as the organizational characteristics that facilitate or constrain nurses’ professional practice, NPEs profoundly influence nurses’ autonomy, the quality of leadership, the availability of resources, the clarity of work processes, and the effectiveness of interprofessional relationships [2]. Recent literature consistently demonstrates that favorable NPEs are associated with improved clinical outcomes [4,7], higher levels of professional satisfaction and lower burnout [5,8,9], and significant reductions in adverse events and mortality [4,6,10].

The ESs constitute one of the most complex, unpredictable, and demanding clinical settings for healthcare delivery, requiring specialized knowledge, sustained concentration, and rapid decision-making, particularly in the care of critically ill patients [11,12,13,14,15].

Several authors have documented the challenges that nurses face daily in ESs. Among the most frequently reported challenges are insufficient nurse staffing levels, both in terms of quantity and specific qualifications or training, as well as breakdowns in communication among professionals. These organizational and contextual factors increase the risk of errors and missed care, which can adversely affect patient safety and the overall quality of care [4,6,8,11,12,13,16,17,18,19,20,21]. Beyond human resource constraints, the physical environment of ESs also represents a critical factor. Overcrowding, often associated with inadequate physical infrastructure, misalignment with care demands, and material resource shortages, constitutes a significant threat to the safety of nursing interventions, thereby increasing risks to the quality and safety of care delivery [12,13,16,18].

Finally, the literature also identifies risk factors related to management and leadership practices in ESs, particularly insufficient preparation and gaps in managerial competencies required to oversee highly complex settings and provide effective support to nursing teams. Such leadership weaknesses may compromise organizational responsiveness, negatively influence the practice environment, and further exacerbate risks to patient safety [4,5,6,8,9,16,18].

One mechanism by which NPEs influence the quality and safety of care is through missed nursing care. Studies conducted across different settings consistently demonstrate that environments characterized by inadequate resources, weak leadership support, limited nurse participation in decision-making, and fragile interprofessional relationships increase the likelihood of omission or delay in the delivery of essential nursing care [22,23,24,25,26]. 

All these constraints lead to the following questions: What characterizes the NPE in ESs? Are nurses able to deliver all necessary care? Are they able to provide safe care in this context?

Despite strong international evidence linking NPEs to quality and patient safety outcomes, most research has focused on inpatient settings, with limited studies specifically addressing ESs. In the Portuguese context, studies based on the RN4CAST framework have mainly examined inpatient and primary healthcare environments, and empirical data on practice environments and care processes in ESs remain limited [3,4,6,12,27,28,29]. Accordingly, available evidence remains limited regarding the characterization of NPEs, organizational conditions, and nurses’ perceptions of job satisfaction, quality and safety of care, as well as the occurrence of safety incidents and missed nursing care in ESs, particularly within the Portuguese context. This gap in the literature supports the need for the present study.

The aim of this study is to describe, across two distinct time points (2018 and 2022), the characteristics of the sample of ES nurses, including sociodemographic, educational, and professional variables, as well as to characterize the organizational context in which they practice, considering both institutional features and specific characteristics of ESs. The study further seeks to describe NPEs and associated professional outcomes, namely job satisfaction, and care-related outcomes, including nurses’ perceptions of quality of care and patient safety. Safety is examined through assessments of overall unit safety, safety culture, and incidents involving both patients and nurses. Finally, the study aims to describe the prevalence of missed nursing care in ESs.

## 2. Materials and Methods

### 2.1. Study Design

To address the previously defined research questions and objectives, an observational, descriptive, cross-sectional study was conducted. At this stage, the focus is exclusively on describing the characteristics of nurses, ESs, and their respective organizations, as well as nurses’ perceptions of their NPEs, professional satisfaction, the quality and safety of care, safety culture, the occurrence of incidents, and the prevalence of missed nursing care. This article, therefore, reports the descriptive phase of a broader research project, while analytical and explanatory models examining the relationships between NPEs and professional and care-related outcomes are being developed in a subsequent phase of the study. This study was reported in accordance with the STROBE guidelines for observational studies.

### 2.2. Context and Sample

This study is part of the Portuguese replication of the international RN4CAST project and was developed based on the analysis of data collected in 2018 and 2022 from nurses working in polyvalent medical-surgical and basic ESs, providing care to adults and children in public, private, and social sector hospitals across mainland Portugal and the Autonomous Regions [30]. Data collection was conducted via an online questionnaire disseminated nationwide with support from the Portuguese Nursing Council (Ordem dos Enfermeiros). The survey link was distributed electronically to nurses across Portugal, regardless of their clinical setting. Participation was voluntary and anonymous.

For the purposes of the present study, only responses from nurses who reported working in ESs were included in the analysis. A non-probability convenience sampling strategy was used, with voluntary participation of eligible professionals, totaling 390 participants in 2018 and 434 in 2022.

Because the questionnaire was disseminated broadly to the national nursing community and information on the total number of nurses working specifically in ESs in Portugal was unavailable, it was not possible to determine the total number of eligible participants or calculate response rates.

Given the survey’s anonymous nature, it was not possible to identify duplicate responses; however, the questionnaire platform limited submissions to one per device, reducing the likelihood of duplicates.

### 2.3. Data Collection Instrument

Data were collected through an online questionnaire that included sociodemographic, educational, and professional information about the nurses. The main variables of interest comprised nursing practice environments, professional satisfaction, perceived quality and safety of care, safety culture, incident occurrence, and missed nursing care. NPEs were assessed using the PES-NWI, which comprises 31 items distributed across five dimensions, in accordance with the interpretation criteria established by the original authors [2,31,32]. The PES-NWI is a widely used instrument internationally and has been translated, culturally adapted, and validated for the Portuguese population [32,33,34]. It has demonstrated adequate psychometric properties in the Portuguese hospital context. Its application in national studies has also enabled the analysis of the relationship between practice environments and care quality and safety [3,28,35]. Professional satisfaction, perceived quality and safety of care, safety culture, incident occurrence, and the prevalence of missed nursing care were assessed using items from the RN4CAST Nurse Survey Instrument (Core Nurse Survey). Professional satisfaction, quality, and safety indicators were measured through specific items using Likert-type scales. Safety culture was evaluated using a 7-item scale, while incident occurrence was measured using a 12-item scale that included events involving both patients and nurses. The prevalence of missed nursing care was assessed using items that asked about the frequency of care left undone or delayed during the last work shift.

### 2.4. Data Analysis

Data were analyzed using descriptive statistics, including measures of central tendency and dispersion for continuous variables, and absolute and relative frequencies for categorical variables. The two data collection periods (2018 and 2022) were examined through parallel descriptive analysis of the results obtained at each time point.

Missing responses were handled using valid cases for each variable. Therefore, only available responses were included in the calculation of descriptive statistics, which explains minor variations in the total number of observations across some variables.

Composite scores for the PES-NWI were calculated as the mean of the items included in each dimension, in accordance with the scoring procedures recommended by the instrument’s authors [2,31]. Following the criteria proposed by Lake and Friese [31], each dimension was interpreted using a cut-off value of 2.5, with mean scores above 2.5 indicating favorable conditions. The overall practice environment was classified according to the number of dimensions exceeding this threshold: environments with 0–2 favorable dimensions were considered unfavorable, those with 3 favorable dimensions were classified as mixed, and those with 4–5 favorable dimensions were considered favorable.

Statistical analyses were performed using IBM SPSS Statistics, Version 31.0 (IBM Corp., Armonk, NY, USA). 

### 2.5. Ethical Considerations

The study was conducted in accordance with the ethical principles of health research, ensuring respect for participants, their dignity, and their professional context. The data analyzed derive from previous data collections conducted within the framework of the Portuguese replication of the RN4CAST study in 2018 and 2022, with the principal investigator’s authorization. The study was approved by the Health Ethics Committee of Universidade Católica Portuguesa (approval nº. 3/2018 and nº. 177/2022).

Participation was voluntary, and anonymity and data confidentiality were strictly ensured. Informed consent was obtained from all participants prior to completion of the questionnaire. All ethical and legal requirements established by the national research team and the international consortium were fully observed.

Data were analyzed anonymously and confidentially, and the results are reported in aggregate to support the dissemination of scientific knowledge.

The authors declare no conflicts of interest.

### 2.6. Data Availability

The data used in this study are not publicly available due to ethical and legal restrictions related to data protection. Data may be made available upon reasonable request to the project’s principal investigators and subject to authorization by the relevant competent authorities.

### 2.7. Use of Generative Artificial Intelligence

Generative artificial intelligence was not used in the study design, data collection, or data analysis. The use of artificial intelligence tools was limited to linguistic and stylistic revision of the manuscript.

## 3. Results

### 3.1. Sociodemographic, Educational, and Professional Characteristics of Participants

The sample comprised nurses working in ESs, including 390 participants in 2018 and 434 in 2022. The majority were female, with a mean age of 37.03 years (SD = 8.25) in 2018 and 38.04 years (SD = 8.56) in 2022.

Regarding educational background, most participants held a bachelor’s degree in nursing (98.7% in 2018 and 99.8% in 2022), and fewer than half were specialist nurses (46.4% in 2018 and 47.2% in 2022). The mean years of experience in the profession, in the institution, and in the service at the time of data collection are presented in Table 1.

### 3.2. Organizational and ES Characteristics

In 2018, most participants worked in the Lisbon and Tejo Valley region (32.3%), followed by the Northern region (27.2%). In 2022, a different distribution was observed, with the highest proportion of participants located in the Northern region, followed by Lisbon and the Tejo Valley. The least represented regions in 2018 were the Algarve (3.6%), the Azores (5.4%), and Madeira (6.4%). In 2022, the Algarve and Madeira showed equal representation (2.3% each), followed by the Azores (3.2%).

The regional distribution of nurses differed between 2018 and 2022, with a statistically significant difference (χ^2^ = 43.502; df = 6; *p* < 0.001). Although regional distributions differed across samples, data were analyzed at the aggregated national level in accordance with the study objective.

Regarding the type of institution, most nurses were employed in National Health Service hospitals, representing 95.6% of participants in 2018 and 97.9% in 2022. Participants were asked to indicate whether their practice context more closely resembled an outpatient (ambulatory) setting or an inpatient unit. Despite working in ESs, nearly half of the nurses perceived their environment as similar to an inpatient setting in both 2018 (48.5%) and 2022 (48.2%).

### 3.3. Nursing Practice Environment

The results of the PES-NWI are presented in Table 2. In 2018, the PES-NWI revealed an overall mean NPE score of 2.28 (SD = 0.41). When ordered from lowest to highest, the dimension with the lowest mean was Staffing and Resource Adequacy (SRA), 1.90 (SD = 0.58); followed by Nurse Participation in Hospital Affairs (NPHA), 2.09 (SD = 0.48); Nurse Manager Ability, Leadership, and Support of Nurses (NMLSN), 2.27 (SD = 0.61); and Nursing Foundations for Quality of Care (NFQC), 2.47 (SD = 0.47). The only dimension with a mean value considered favorable, according to Lake’s classification [2], was Collegial Nurse–Physician Relations (CNPR), with a mean of 2.76 (SD = 0.46).

In 2022, the overall mean NPE score was also classified as unfavorable at 2.23 (SD = 0.42), according to Lake’s classification [2]. Again, when ordered from lowest to highest, the lowest mean was observed in Staffing and Resource Adequacy (SRA), 1.85 (SD = 0.57); followed by Nurse Participation in Hospital Affairs (NPHA), 2.00 (SD = 0.51); Nurse Manager Ability, Leadership, and Support of Nurses (NMLSN), 2.26 (SD = 0.60); and Nursing Foundations for Quality of Care (NFQC), 2.42 (SD = 0.48). In the 2022 sample, the only dimension considered favorable was Collegial Nurse–Physician Relations (CNPR), with a score of 2.75 (SD = 0.49).

Based on the criteria proposed by Lake and Friese [31], most nurses in both samples were working in environments classified as unfavorable. In 2018, 69.7% of respondents were classified as working in unfavorable practice environments, 14.4% in mixed environments, and 15.9% in favorable environments. In 2022, 71.2% of respondents were classified as working in unfavorable environments, 16.4% as in mixed environments, and 12.4% as in favorable environments.

### 3.4. Job Satisfaction, Work Environment, Intention to Leave, and Institutional Recommendation

The results regarding professional satisfaction, work environment, intention to leave, and institutional recommendation are presented in Table 3. Nurses evaluated their satisfaction with their current role using a four-point scale ranging from “very dissatisfied” to “very satisfied.” Mean values indicate generally low levels of satisfaction in both 2018 (M = 2.31; SD = 0.85) and 2022 (M = 2.26; SD = 0.88). In 2018, most nurses reported being moderately satisfied (41.3%) or slightly dissatisfied (33.8%) with their role. Only 4.9% reported being very satisfied, while 20.0% indicated being very dissatisfied. A similar distribution was observed in 2022, with 35.9% reporting moderate satisfaction and 35.5% slight dissatisfaction. At the negative extreme, 22.4% reported being very dissatisfied, whereas only 6.2% reported being very satisfied.

Regarding perceptions of the work environment, also assessed on a four-point scale from “poor” to “excellent,” mean values fall in the lower range in both 2018 (M = 1.94; SD = 0.74) and 2022 (M = 1.91; SD = 0.79). In 2018, most participants classified the work environment as fair (47.4%), followed by good (22.1%) and poor (29.7%), with only 0.8% considering it excellent. A comparable pattern was observed in 2022, with 43.5% rating the environment as fair, 33.6% as poor, and only 2.1% as excellent.

When asked about the possibility of leaving the organization in the following year due to job dissatisfaction, more than half of the nurses responded affirmatively in both 2018 (53.8%) and 2022 (56.9%). Among those considering leaving the organization, 33.3% in 2018 reported contemplating leaving the profession, compared with 41.3% in the 2022 sample. Regarding the perceived ease of finding another acceptable nursing position, 28.2% of nurses in 2018 considered it very difficult, while 4.6% reported it would be very easy. In the 2022 sample, 18.9% considered it very difficult, whereas 6.7% considered it very easy to find another acceptable position. Concerning colleagues’ recommendations of the institution as a workplace, only 7.2% of nurses in 2018 reported that they would definitely recommend the organization, while 9.0% stated that they would definitely not recommend it. In the 2022 sample, 4.6% reported that they would definitely recommend the organization, whereas 13.6% reported that they would definitely not recommend it. When asked to recommend the institution as a place to receive care to friends or family members, most nurses indicated they would probably recommend their hospital in both 2018 (56.2%) and 2022 (51.8%). In 2018, 13.8% reported that they would definitely recommend their institution, whereas 6.2% reported that they would definitely not recommend it. In the 2022 sample, 9.0% reported that they would definitely not recommend the institution, while 12.0% reported that they would definitely recommend it.

### 3.5. Quality and Safety of Care

#### 3.5.1. Quality of Care

The quality of nursing care provided in the service was assessed using a single item on a four-point scale from “poor” to “excellent.” The mean quality scores were moderate, falling between the categories “fair” and “good” in both 2018 (M = 2.44; SD = 0.77) and 2022 (M = 2.28; SD = 0.80). In 2018, most nurses classified the quality of care as good (44.4%) or fair (37.7%), while 12.1% rated it as poor and 5.6% as excellent. In 2022, the highest proportions were observed in the fair (37.2%) and good (40.9%) categories, followed by poor (18.9%) and excellent (3.0%) (Table 4).

The perceived change in quality of care over the previous year was assessed through a direct question using a three-point ordinal scale (1 = worsened; 2 = remained the same; 3 = improved), asking nurses to indicate whether, in their view, the quality of care provided to patients in their hospital had worsened, remained the same, or improved during the last year. Mean scores indicate that nurses’ perceptions fell between “worsened” and “remained the same” in both samples, with slightly higher values in 2018 (M = 1.71; SD = 0.70) than in 2022 (M = 1.48; SD = 0.64). In 2018, 42.8% reported that quality had worsened, and 13.6% reported that it had improved. In 2022, the majority (59.7%) indicated that quality had worsened, whereas only 7.8% reported improvement (Table 4).

Trust in hospital management was assessed through a single item measured on a four-point Likert scale, ranging from “not at all confident” to “very confident”, asking nurses to rate their level of confidence in how hospital management would address problems related to patient care that they had reported. Mean confidence scores were low, corresponding to values between “not confident” and “somewhat confident” (2018: M = 1.97, SD = 0.70; 2022: M = 1.82, SD = 0.72). In 2018, 80.0% of nurses reported being little or not at all confident, compared with 82.9% in the 2022 sample. The proportion of respondents who reported being “very confident” was residual in both years (Table 4).

#### 3.5.2. Safety of Care

The safety of the ES was assessed using a five-point scale ranging from “failing” to “excellent.” In both samples, the mean safety scores were low, falling between the categories “poor” and “acceptable”, suggesting suboptimal perceptions of safety (2018: M = 2.50, SD = 1.07; 2022: M = 2.39, SD = 1.06). In 2018, the most frequent classification was “acceptable” (39.3%), whereas only 1.0% of participants rated safety as “excellent” and 18.3% as “poor.” In 2022, a similar pattern was observed, with 34.9% of nurses rating safety as “acceptable,” and the proportion of “excellent” ratings remaining residual (0.9%) (Table 5).

#### 3.5.3. Safety Culture

A descriptive analysis of nurses’ perceptions of safety culture was conducted in 2018 and 2022, based on seven items adapted from the *Hospital Survey on Patient Safety Culture* developed by the Agency for Healthcare Research and Quality [36], which is widely used in international research to assess safety climate [37]. These items address dimensions such as freedom to question hierarchical decisions, punitive responses to errors, communication during care transitions, feedback following event reporting, and management commitment to patient safety. Responses were measured on a five-point Likert scale (1 = strongly disagree; 5 = strongly agree). Overall, safety culture values were around the midpoint of the scale, with a mean of 2.97 (SD = 0.46) in 2018 and 2.81 (SD = 0.70) in 2022. In 2018, 26.9% of nurses agreed or strongly agreed that mistakes are held against staff, which may indicate a potentially punitive culture. Regarding freedom to question decisions made by superiors, 44.3% in 2018 and 42.0% in 2022 disagreed or strongly disagreed that such freedom existed. Concerning management commitment to safety as a top priority, approximately half of participants disagreed or strongly disagreed (51.5% in 2018; 48.5% in 2022). Conversely, most nurses reported that their unit discusses ways to prevent errors from recurring (50.5% in 2018; 52.2% in 2022 agreed or strongly agreed), although the proportion reporting structured feedback after event reporting was low in both samples (30.7% in 2018; 29.7% in 2022). Regarding care transitions, 38.9% in 2018 and 35.3% in 2022 agreed that important information is often lost during shift changes, and 32.9% and 29.2%, respectively, acknowledged that aspects of care are neglected during inter-unit transfers, indicating potential weaknesses in continuity and patient safety. Detailed results are presented in Appendix A.

#### 3.5.4. Incident Occurrence

The frequency of incident occurrence was assessed using a seven-point scale ranging from 0 (“never”) to 6 (“every day”). Overall, the mean incident frequency was 1.76 (SD = 1.03) in 2018 and 2.80 (SD = 1.04) in 2022. Considering the scale anchors, the 2018 mean falls between “a few times a year or less” and “once a month or less,” suggesting a perception of sporadic incident occurrence. In 2022, the mean approached the “a few times a month” category, indicating a different perceived frequency of incidents. These findings indicate differences in the perceived frequency of incidents between the two samples, with similar variability observed in both.

Regarding patient-related incidents, most responses were concentrated in the lower-frequency categories (“a few times a year or less” and “once a month or less”) in both years. However, higher-frequency categories were reported more often in the 2022 sample. For patient falls with injury, responses of “a few times a month” were higher in the 2022 sample (11.3%) compared with 2018 (5.4%), and responses of “weekly or more frequently” were also higher (9.4% vs. 3.1%). For pressure ulcers, the categories “a few times a week” and “every day” were higher in 2022 (8.8%) than in 2018 (5.2%).

Among nurse-related incidents, verbal aggression from patients and/or relatives was particularly frequent, with 46.0% of nurses reporting weekly or daily occurrences in 2018 and 53.8% in 2022. Physical aggression by patients/relatives was also higher in the monthly-or-more categories, increasing from 16.4% to 22.8%. Work-related physical injuries showed a relevant distribution in higher-frequency categories, including 5.2% of “every day” responses in 2022.

Detailed descriptive results by type and full item distributions are presented in Appendix B.

### 3.6. Missed Nursing Care

With regard to missed nursing care, 13 nursing activities potentially left undone due to lack of time were assessed, namely: adequate patient surveillance; treatments and procedures; preparing patients and families for discharge; care planning; frequent patient repositioning; oral hygiene; pain management; developing or updating nursing care plans/options; educating patients and families; proper documentation of nursing care; skin care; comforting and communicating with patients; and administering medication on schedule.

Only a small proportion of nurses reported completing all required care during their last shift in both 2018 (6.3%) and 2022 (6.7%). The mean number of missed nursing care activities was 5.37 (SD = 3.30) in 2018 and 5.90 (SD = 3.67) in 2022 (Table 6).

When analyzed by shift, the afternoon shift in 2018 had the highest mean number of missed care activities, followed by the morning and night shifts. In 2022, the highest mean number of missed care activities was reported for the morning shift, followed by the afternoon and night shifts.

Regarding the types of missed care, the most frequently reported in both periods included comforting/talking with patients, educating patients and families, adequate documentation of nursing care, oral hygiene, and developing or updating nursing care plans. In 2022, adequate patient surveillance was also among the most frequently missed care activities.

Items related to missed nursing care were presented only to nurses who, in the questionnaire, identified their practice context as more like an inpatient setting; therefore, the number of responses analyzed in this domain is lower than the total sample size.

The detailed descriptive results of missed nursing care, including its distribution by type of care and by shift, are presented in Appendix C.

## 4. Discussion

The discussion interprets the study findings in light of the available scientific evidence, focusing on the patterns observed in the two independent samples within Portuguese ESs. Adopting a descriptive approach, the analysis focuses on NPEs and on professional and care-related outcomes, highlighting relevant implications for clinical practice, management, and nursing care safety.

The organizational context was characterized by a description of the main features of the institutions and ESs in which nurses were practicing. A greater concentration of nurses was observed in institutions in the North and in the Lisbon and Tejo Valley regions, where the main national referral centers are located, as well as a predominance of the public sector, reinforcing the public sector’s central role in providing emergency and critical care. However, nearly half of the nurses reported that their work settings more closely resembled inpatient units than the theoretical model of ESs, which are designed to provide predominantly ambulatory, short-term care focused on clinical stabilization and appropriate patient referral [30,38,39]. This perception may be explained by structural and organizational factors, such as the intensive use of observation areas and the prolonged length of stay of patients in ESs, often associated with chronic overcrowding, difficulties in transferring patients to inpatient units, and limitations within the continuing care network. Many patients remain under nurses’ care for extended periods, requiring care typically delivered in inpatient settings, often in conditions that do not ensure adequate privacy, comfort, or structural suitability. This discrepancy between the normative model and everyday practice may be associated with increased workload and the perception of an inadequate practice environment, with potential implications for the quality, safety, and humanization of care, a phenomenon widely documented in the national literature [38]. These findings help contextualize the overall unfavorable perception of NPEs identified in this study.

The results of this study indicate a predominantly unfavorable pattern of NPEs in Portuguese ESs, with around 70% of nurses in both samples (2018 and 2022) working in environments classified as unfavorable [31].

The dimension related to staffing and resource adequacy was the most negatively evaluated, suggesting a widespread perception of insufficient resources to meet care demands. This finding aligns with other studies that report high levels of professional dissatisfaction, intentions to leave the organization, negative perceptions of care quality, and frequent omissions of essential nursing care. International literature supports these findings, identifying resource adequacy as one of the most fragile dimensions of NPEs and associating lower staffing levels with increased mortality, higher rates of adverse events, and greater risk of missed nursing care [10,40,41,42,43,44,45]. In the specific context of ESs, shortages of human and material resources represent a widely recognized structural challenge and may also be relevant in the Portuguese context [46,47,48].

Nurses’ participation in hospital affairs ranked second among the most negatively rated dimensions, indicating a limited sense of influence in organizational decision-making. This fragility appears to be associated with low levels of trust in institutional leadership and is accompanied by perceptions of unresponsive leadership, insufficient feedback, and limited recognition of nurses’ professional contributions. These findings are consistent with previous studies that identify participatory governance as one of the weakest domains of NPEs, with its limitations associated with lower job satisfaction and a higher intention to leave the profession [3,35,49,50]. Similarly, the dimension of nurse manager ability, leadership, and support of nurses was rated unfavorably, contrasting with recent evidence showing that transformational leadership styles, visible leadership, and effective managerial support are associated with higher engagement, greater team resilience, and improved safety and job satisfaction outcomes, particularly in high-demand settings such as ESs [47,51].

In contrast to these identified weaknesses, collegial nurse–physician relations were the only dimension rated favorably, suggesting that interprofessional collaboration may be a relevant protective factor, even in adverse organizational contexts. The literature widely recognizes this dimension as a key determinant of patient safety, linking it to lower error rates, improved clinical outcomes, and higher satisfaction among both professionals and patients [52,53,54]. It is also important to note the slight difference observed in the overall mean score for NPEs between the two samples (2018 and 2022). Previous studies have highlighted pre-existing weaknesses within healthcare organizations, particularly regarding staffing levels, emotional burden, and professional burnout [10,51,55,56]. Although the numerical difference observed in this study is modest, it may reflect differences in nurses’ perceptions of practice environments between the two samples and is consistent with structural and organizational weaknesses, with potential implications for professional satisfaction and patient safety. In summary, the findings reveal multidimensional challenges in NPEs within Portuguese ESs, highlighting the importance of structural interventions to strengthen resources, leadership, and nurses’ participation in governance as essential conditions for improving safety, quality of care, and the sustainability of these services.

Next, nurses’ professional outcomes are examined, with a particular focus on job satisfaction alongside NPEs. The findings indicate low levels of professional satisfaction in both samples, observed alongside globally unfavorable practice environments. Satisfaction with career choice, current position, and work environment was limited in 2018 and 2022, with high levels of intention to leave the organization and, in 2022, the profession itself. These results are consistent with the international literature, which links unfavorable practice environments to higher emotional exhaustion, reduced organizational commitment, and increased turnover intention [8,55,56,57,58]. At the same time, a paradox emerged regarding organizational prestige: nurses were more willing to recommend the institution as a place to receive care than as a place to work. This pattern has been described in the literature as being associated with fragile organizational climates, increased turnover risk, and reduced professional attractiveness [18]. Taken together, these findings highlight the importance of targeted organizational interventions to improve NPEs, a key factor in enhancing staff retention and ensuring the quality and safety of care.

Mixed perceptions were observed regarding the quality of nursing care in Portuguese ESs. Although more than half of the nurses rated the quality of care as “good” or “excellent” in 2022 (and approximately 50% in 2018), a higher proportion of nurses in 2022 reported that care quality had worsened in the previous year (59.7% vs. 42.8%). This apparent paradox may reflect the coexistence of differing perceptions of care quality among nurses and contextual factors described in the literature, such as unfavorable practice environments and increasing workload pressures, particularly in the 2022 sample. Furthermore, persistently low confidence in leadership’s ability to address reported problems may contribute to the perception that organizational improvement is limited. International evidence suggests that settings characterized by limited leadership presence, inadequate staffing, and excessive workload contribute to discrepancies between nurses’ perceptions of their individual care performance and their overall assessment of institutional care quality [4,59,60].

The assessment of overall service safety reveals predominantly negative perceptions among nurses working in ESs, with lower values observed in the 2022 sample. Only a minority rated the safety of their service as excellent, suggesting potential vulnerabilities within the care environment. This perception may be associated with factors described in the literature, such as unfavorable NPEs, organizational constraints, and challenging working conditions, further highlighting potential concerns regarding patient safety in these settings [10,47].

Findings on safety culture indicate organizational weaknesses in ESs, particularly in leadership and institutional support. Nurses’ low confidence in management’s ability to address reported problems, combined with the perception of limited feedback following incident reporting, suggests constraints in the development of a learning-oriented culture. These vulnerabilities are evident in the limited freedom to question hierarchical decisions, perceptions of punitive responses to errors, and a lack of clear visibility into managerial actions, thereby suggesting limitations in how patient safety is prioritized. Although nurses acknowledge opportunities to discuss strategies to prevent the recurrence of errors, the absence of structured, systematic feedback may limit the development of mature safety cultures. International evidence has shown that unfavorable NPEs, coupled with less visible or less responsive leadership, are associated with higher rates of adverse events, lower error reporting, and increased professional dissatisfaction [42,43,61]. In contrast, settings characterized by adequate staffing levels, visible leadership, and effective managerial support for nurses are consistently associated with better safety outcomes. Furthermore, the weaknesses identified in continuity of care, particularly the loss of information during shift handovers and inter-unit transfers, are consistent with well-documented safety risks in highly complex environments such as ESs [17].

The analysis of incident occurrence indicates differences in the perceived frequency of adverse events between the two samples, involving both patients and professionals. While in 2018 incidents were predominantly described as sporadic events (“a few times per year or less”), in 2022 the mean was higher, reaching “once a month or less,” which may reflect differences in nurses’ perceptions or reporting of incidents between the two samples. Among patient-related incidents, medication administration errors, pressure injuries, falls, and healthcare-associated infections were particularly prominent, with higher frequencies reported in the 2022 sample. Regarding professionals, verbal and physical aggression by patients and/or family members stood out, highlighting potential risks to occupational safety and team satisfaction. Recent literature has reported associations between increased workload, insufficient resources, and institutional fatigue and a higher prevalence of errors, adverse events, and occupational incidents among healthcare professionals; these findings align with those of the present study [62,63,64].

As previously noted, missed nursing care was assessed only among nurses who described their practice environment as more akin to an inpatient setting. The findings indicate a high frequency of missed nursing care within this subgroup. Similar patterns were observed in both samples (2018 and 2022), with higher mean values in the 2022 sample. Only approximately 6% of nurses reported being able to deliver all required care during their last shift, a notably low and clinically significant proportion. Across both time points, the most frequently missed care activities were autonomous nursing interventions, such as providing comfort and engaging in dialogue with patients, educating patients and families, and developing or updating nursing care plans, areas that require time, clinical reflection, and direct interaction. The omission of these interventions is widely described as an indirect indicator of safety risk, as it may compromise continuity of care, humanization, and shared decision-making. In contrast, interdependent interventions, such as medication administration and technical procedures, tended to be preserved, in line with the existing literature [14,65,66]. Although the underlying causes were not directly examined in this study, existing evidence suggests an association between missed nursing care and insufficient staffing and material resources, ES overcrowding, organizational challenges, and organizational culture and leadership. These findings highlight the relevance of strategies to strengthen NPEs as a means to address missed nursing care in ESs [65,67,68,69,70].

This study has several limitations that should be considered when interpreting the findings. Its descriptive design, based on data collected at two independent time points (2018 and 2022), does not allow causal inferences or the follow-up of individual participants over time. Therefore, differences between the results should be interpreted with caution and not considered temporal trends. The use of self-reported data based on nurses’ perceptions may introduce subjective bias and does not reflect objective clinical or organizational performance indicators. Additionally, the study used a non-probability convenience sampling strategy, which may limit the sample’s representativeness. The questionnaire was disseminated nationwide through the Portuguese Nursing Council (Ordem dos Enfermeiros), reaching nurses from different clinical contexts. As national data on the number of nurses working specifically in ESs in Portugal were unavailable, it was not possible to determine the total number of eligible participants or calculate response rates. Regional heterogeneity between samples may influence comparability. However, the data were analyzed at the aggregated national level, in line with the study’s overall objective, which may limit comparability of results across regions. Although the present study is descriptive, this approach was intentional and represents the first step in a multi-phase research design. Establishing a comprehensive national characterization of NPEs in ESs was considered an important step before advancing to analytical and explanatory modeling. Furthermore, the study focuses primarily on nursing-related variables and does not include broader organizational and management-related factors, which may also play a relevant role in shaping the observed outcomes. In addition, integrating organizational and management-related variables may enhance understanding of the complexity of nursing practice environments and their impact on professional and care-related outcomes.

Despite these limitations, the study offers relevant contributions across multiple domains. At the level of care delivery, the findings highlight the importance of organizational conditions that enable nurses to provide comprehensive, safe, and person-centered care, which may contribute to reducing the omission of essential interventions. In terms of management and governance, the results support the importance of institutional policies that focus on adequate staffing and material resources, strengthen nursing leadership, and promote participatory governance models. Regarding education and professional development, the findings highlight the relevance of investing in competencies in leadership, patient safety, and complexity management in emergency care settings. Finally, from a research perspective, this study provides a useful foundation for future analyses exploring the relationships among NPEs, professional outcomes, and care-related outcomes, as well as for longitudinal and analytical studies aimed at deepening understanding of the mechanisms underlying the identified phenomena.

## 5. Conclusions

This study showed that NPEs in Portuguese ESs were globally unfavorable in 2018 and 2022, with limitations in staffing and material resources, leadership, and nurses’ involvement in organizational governance. Although collegial nurse–physician relations were positive across both data collection periods, the findings reveal low job satisfaction, high intention to leave the organization, and, in 2022, negative perceptions of care quality and safety, higher reported incident frequencies, and a high frequency of missed nursing care among nurses working in inpatient-like contexts within ESs, particularly during autonomous nursing interventions. In line with the defined objectives, these findings enable an integrated interpretation of NPEs, professional outcomes, and care-related outcomes in ESs.

Overall, the data suggest that the identified structural and organizational weaknesses may be associated with nurses’ professional experience and care-related outcomes. Strengthening NPEs through policies focused on adequate resource allocation, the development of more participative leadership models, and the recognition of nurses’ contributions to decision-making processes may be an important strategy to support care quality and safety, support nurse retention, and sustain nursing teams in this highly demanding setting.

This study makes several relevant contributions to knowledge regarding ESs in Portugal. First, it provides a comprehensive and up-to-date characterization of NPEs, integrating professional and care-related outcomes to offer a coherent, global understanding of the practice context. The descriptive analysis of two distinct time points (2018 and 2022) is particularly relevant, as it highlights challenges in one of the most demanding sectors of the healthcare system. Furthermore, the specific focus on ESs, which are often underrepresented in national research, enhances the originality of this work. The use of validated, internationally recognized instruments, such as the PES-NWI, together with the assessment of critical dimensions, including safety culture, incident occurrence, and missed nursing care, supports the robustness and international comparability of the findings. Collectively, this study provides relevant empirical evidence to inform clinical practice, healthcare management, and organizational policy development to support improvements in NPEs, patient safety, and quality of care in ESs. The findings also provide a robust empirical basis for subsequent analytical studies that examine potential pathways among practice environments, nurse outcomes, and patient safety indicators in emergency care settings.

## Figures and Tables

**Table 1:** Nurses’ years of experience in the profession, in the institution, and in the service (2018 vs. 2022).

Experience	2018		2022	
	M	SD	M	SD
In the profession	14.20	8.34	14.81	8.44
In the institution	12.28	8.44	12.04	9.41
In the service	8.84	6.63	9.81	8.29

M—mean; SD—standard deviation.

**Table 2:** NPEs in Portuguese ESs (2018 vs. 2022).

	2018			2022		
	*n*	M	SD	*n*	M	SD
Global Scale	390	2.28	0.41	434	2.23	0.42
NPHA	390	2.09	0.48	434	2.00	0.51
NFQC	390	2.47	0.47	434	2.41	0.48
NMLSN	390	2.27	0.61	434	2.26	0.60
SRA	390	1.90	0.58	434	1.85	0.57
CNPR	390	2.76	0.46	434	2.75	0.49

M—mean; SD—standard deviation; NPHA—Nurse Participation in Hospital Affairs; NFQC—Nursing Foundations for Quality of Care; NMLSN—Nurse Manager Ability, Leadership, and Support of Nurses; SRA—Staffing and Resource Adequacy; CNPR—Collegial Nurse–Physician Relations.

**Table 3:** Professional satisfaction, intention to leave, and institutional recommendation (2018 vs. 2022).

Indicator	Scale	2018		2022	
		*n*	%	*n*	%
**Job satisfaction**	Very dissatisfied	78	20.0	97	22.4
	Slightly dissatisfied	132	33.8	154	35.5
	Moderately satisfied	161	41.3	156	35.9
	Very satisfied	19	4.9	27	6.2
	**Total**	**390**	**100**	**434**	**100**
**Work environment**	Poor	116	29.7	146	33.6
	Fair	185	47.4	189	43.5
	Good	86	22.1	90	20.7
	Excellent	3	0.8	9	2.1
	**Total**	**390**	**100**	**434**	**100**
**Intention to leave**	Yes	210	53.8	247	56.9
	No	180	46.2	187	43.1
	**Total**	**390**	**100**	**434**	**100**
**Perceived difficulty in finding another job**	Very difficult	110	28.2	82	18.9
	Relatively difficult	163	41.8	160	36.9
	Relatively easy	99	25.4	163	37.6
	Very easy	18	4.6	29	6.7
	**Total**	**390**	**100**	**434**	**100**
**Recommendation to colleagues (workplace)**	Definitely not	35	9.0	59	13.6
	Probably not	146	37.4	172	39.6
	Probably yes	181	46.4	183	42.2
	Definitely yes	28	7.2	20	4.6
	**Total**	**390**	**100**	**434**	**100**
**Recommendation to colleagues and family (place to receive care)**	Definitely not	24	6.2	39	9.0
	Probably not	93	23.8	118	27.2
	Probably yes	219	56.2	225	51.8
	Definitely yes	54	13.8	52	12.0
	**Total**	**390**	**100**	**434**	**100**

**Table 4:** Quality of Care as Perceived by Nurses (2018 vs. 2022).

Indicator	Scale	2018		2022	
		*n*	%	*n*	%
**Quality of care**	Poor	47	12.1	82	18.9
	Fair	147	37.7	161	37.2
	Good	173	44.4	177	40.9
	Excellent	22	5.6	13	3.0
	**Total**	**390**	**100**	**433**	**100**
**Perceived change in quality (past year)**	Worsened	167	42.8	259	59.7
	Remained the same	170	43.6	141	32.5
	Improved	53	13.6	34	7.8
	**Total**	**390**	**100**	**434**	**100**
**Confidence in hospital management**	Not confident at all	94	24.1	156	35.9
	Slightly confident	218	55.9	204	47.0
	Confident	73	18.7	71	16.4
	Very confident	5	1.3	3	0.7
	**Total**	**390**	**100**	**434**	**100**

**Table 5:** ES Safety Classification (2018 vs. 2022).

ES Safety Classification	2018		2022	
	*n*	%	*n*	%
Failing	98	25.2	118	27.3
Poor	71	18.3	98	22.6
Acceptable	153	39.3	151	34.9
Very Good	63	16.2	62	14.3
Excellent	4	1.0	4	0.9
**Total**	**389**	**100**	**433**	**100**

**Table 6:** Missed Nursing Care (2018 vs. 2022).

Year	N	Minimum	Maximum	M	SD
2018	189	0	13	5.37	3.30
2022	209	0	13	5.90	3.67

M—mean; SD—standard deviation.

## Data Availability

The data presented in this study are not publicly available due to ethical and privacy restrictions, as they contain information collected from healthcare professionals under confidentiality agreements.

## Public Involvement Statement

No public involvement in any aspect of this research.

## Guidelines and Standards Statement

This manuscript was drafted in accordance with the STROBE guidelines (Strengthening the Reporting of Observational Studies in Epidemiology) for observational research.

## Abbreviations

The following abbreviations are used in this manuscript:

ES	Emergency Service
NPE	Nursing Practice Environment

## Appendix A

This appendix presents the detailed descriptive results of the Safety Culture scale (Table A1).

**Table A1:** Distribution of Responses to the Safety Culture Scale Items (2018 vs. 2022).

Item	Scale	2018		2022	
		*n*	%	*n*	%
**Staff feel that their mistakes are held against them**	Strongly disagree	33	8.5	60	13.8
	Disagree	142	36.4	142	32.7
	Neither agree nor disagree	110	28.2	130	30.0
	Agree	85	21.8	92	21.2
	Strongly agree	20	5.1	10	2.3
	**Total**	**390**	**100**	**434**	**100**
**Staff feel free to question the decisions or actions of those in authority**	Strongly disagree	38	9.7	51	11.8
	Disagree	135	34.6	131	30.2
	Neither agree nor disagree	87	22.3	122	28.1
	Agree	123	31.5	121	27.9
	Strongly agree	7	1.8	9	2.1
	**Total**	**390**	**100**	**434**	**100**
**In this unit, we discuss ways to prevent errors from happening again**	Strongly disagree	41	10.5	30	6.9
	Disagree	88	22.6	103	23.8
	Neither agree nor disagree	64	16.4	74	17.1
	Agree	172	44.1	203	46.9
	Strongly agree	25	6.4	23	5.3
	**Total**	**390**	**100**	**433**	**100**
**We receive feedback about changes implemented based on event reports**	Strongly disagree	60	15.4	68	15.7
	Disagree	114	29.2	123	28.3
	Neither agree nor disagree	96	24.6	114	26.3
	Agree	107	27.4	118	27.2
	Strongly agree	13	3.3	11	2.5
	**Total**	**390**	**100**	**434**	**100**
**Actions taken by hospital/organizational management demonstrate that patient safety is a top priority**	Strongly disagree	70	17.9	98	22.7
	Disagree	131	33.6	111	25.8
	Neither agree nor disagree	93	23.8	113	26.2
	Agree	79	20.3	95	22.0
	Strongly agree	17	4.4	14	3.2
	**Total**	**390**	**100**	**431**	**100**
**Important patient care information is often lost during shift changes**	Strongly disagree	36	9.2	37	8.5
	Disagree	140	35.9	161	37.1
	Neither agree nor disagree	62	15.9	83	19.1
	Agree	130	33.3	144	33.2
	Strongly agree	22	5.6	9	2.1
	**Total**	**390**	**100**	**434**	**100**
**Aspects of patient care are sometimes neglected during transfers between units**	Strongly disagree	34	8.7	35	8.1
	Disagree	160	41.0	175	40.3
	Neither agree nor disagree	67	17.2	97	22.4
	Agree	110	28.3	120	27.6
	Strongly agree	18	4.6	7	1.6
	**Total**	**389**	**100**	**434**	**100**

## Appendix B

This appendix presents the detailed descriptive results of Incident Occurrence, including the distribution of responses for each item (Table A2).

**Table A2:** Distribution of Responses to the Incident Occurrence Scale Items (2018 vs. 2022).

Item	Scale	2018		2022	
		*n*	%	*n*	%
**Administration of wrong medications, incorrect doses, or medications given at the wrong time**	Never	93	23.8	104	24.0
	A few times per year or less	194	49.7	204	47.1
	Once per month or less	32	8.2	29	6.7
	A few times per month	30	7.7	41	9.5
	Once per week	14	3.6	21	4.8
	A few times per week	18	4.6	25	5.8
	Every day	9	2.3	9	2.1
	**Total**	**390**	**100**	**433**	**100**
**Pressure injuries developed after admission**	Never	102	26.2	98	22.6
	A few times per year or less	166	42.6	185	42.6
	Once per month or less	49	12.6	54	12.4
	A few times per month	37	9.5	46	10.6
	Once per week	16	4.1	13	3.0
	A few times per week	19	4.9	33	7.6
	Every day	1	0.3	5	1.2
	**Total**	**390**	**100**	**434**	**100**
**Patient falls with associated injuries**	Never	106	27.2	75	17.3
	A few times per year or less	208	53.5	214	49.3
	Once per month or less	42	10.8	55	12.7
	A few times per month	21	5.4	49	11.3
	Once per week	10	2.6	21	4.8
	A few times per week	2	0.5	16	3.7
	Every day	0	0	4	0.9
	**Total**	**389**	**100**	**434**	**100**
**Urinary tract infections**	Never	77	19.9	76	17.7
	A few times per year or less	140	36.3	156	36.3
	Once per month or less	57	14.8	55	12.8
	A few times per month	60	15.5	67	15.6
	Once per week	20	5.2	28	6.5
	A few times per week	26	6.7	36	8.4
	Every day	6	1.6	12	2.8
	**Total**	**386**	**100**	**430**	**100**
**Bloodstream infections**	Never	124	32.4	125	29.3
	A few times per year or less	155	40.5	184	43.1
	Once per month or less	53	13.8	52	12.2
	A few times per month	22	5.7	38	8.9
	Once per week	15	3.9	13	3.0
	A few times per week	8	2.1	11	2.6
	Every day	6	1.6	4	0.9
	**Total**	**383**	**100**	**427**	**100**
**Pneumonia**	Never	95	24.7	103	24.0
	A few times per year or less	118	30.6	147	34.3
	Once per month or less	57	14.8	58	13.5
	A few times per month	50	13.0	67	15.6
	Once per week	28	7.3	24	5.6
	A few times per week	29	7.5	24	5.6
	Every day	8	2.1	6	1.4
	**Total**	**385**	**100**	**429**	**100**
**Complaints from patients and families**	Never	46	12.1	26	6.1
	A few times per year or less	100	26.4	119	27.8
	Once per month or less	46	12.1	35	8.2
	A few times per month	49	12.9	75	17.5
	Once per week	30	7.9	23	5.4
	A few times per week	55	14.5	72	16.8
	Every day	53	14.0	78	18.2
	**Total**	**379**	**100**	**428**	**100**
**Verbal abuse toward nurses by patients and/or family members**	Never	10	2.7	3	0.7
	A few times per year or less	59	15.7	48	11.5
	Once per month or less	32	8.5	34	8.2
	A few times per month	60	16.0	68	16.3
	Once per week	42	11.2	39	9.4
	A few times per week	94	25.0	127	30.5
	Every day	79	21.0	97	23.3
	**Total**	**376**	**100**	**416**	**100**
**Verbal abuse toward nurses by staff members**	Never	97	25.7	77	18.6
	A few times per year or less	129	34.1	171	41.2
	Once per month or less	50	13.2	42	10.1
	A few times per month	43	11.4	58	14.0
	Once per week	28	7.4	25	6.0
	A few times per week	22	5.8	29	7.0
	Every day	9	2.4	13	3.1
	**Total**	**378**	**100**	**415**	**100**
**Physical assault toward nurses by patients and/or family members**	Never	79	21.2	66	16.5
	A few times per year or less	157	42.1	180	45.1
	Once per month or less	49	13.1	48	12.0
	A few times per month	31	8.3	48	12.0
	Once per week	23	6.2	14	3.5
	A few times per week	27	7.2	30	7.5
	Every day	7	1.9	13	3.3
	**Total**	**373**	**100**	**399**	**100**
**Physical assault toward nurses by staff members**	Never	265	71.2	272	67.8
	A few times per year or less	69	18.5	97	24.2
	Once per month or less	20	5.4	14	3.5
	A few times per month	10	2.7	10	2.5
	Once per week	3	0.8	3	0.7
	A few times per week	2	0.5	5	1.2
	Every day	3	0.8	0	0.0
	**Total**	**372**	**100**	**401**	**100**
**Work-related physical injuries among nurses**	Never	29	7.6	29	6.8
	A few times per year or less	149	38.9	154	36.3
	Once per month or less	60	15.7	66	15.6
	A few times per month	63	16.4	91	21.5
	Once per week	40	10.4	25	5.9
	A few times per week	30	7.8	37	8.7
	Every day	12	3.1	22	5.2
	**Total**	**383**	**100**	**424**	**100**

## Appendix C

This appendix also presents the descriptive results of missed nursing care by shift (morning, afternoon, and night), expressed as mean and standard deviation, for the 2018 and 2022 data collection periods (Table A3).

**Table A3:** Missed nursing care by Shift (2018 vs. 2022).

Year	Shift	N	Minimum	Maximum	M	SD
**2018**	Morning	67	0	13	5.31	3.53
	Afternoon	50	1	13	5.56	3.24
	Night	72	0	13	5.28	3.17
**2022**	Morning	60	0	13	6.53	3.75
	Afternoon	70	0	13	5.71	3.38
	Night	79	0	13	5.59	3.82

The present appendix also presents the distribution of missed nursing care due to time constraints, as perceived by nurses, for the 2018 and 2022 data collection periods (Table A4).

**Table A4:** Missed nursing care by type of care (2018 vs. 2022).

Missed Nursing Care	2018		2022	
	N	%	N	%
Adequate patient surveillance	88	46.6	136	65.1
Treatments and procedures	17	9.0	22	10.5
Preparing patients and families for discharge	90	47.6	92	44.0
Care planning	74	39.2	86	41.1
Repositioning patients and families for discharge	55	29.1	70	33.5
Oral hygiene	98	51.9	112	53.6
Pain management	32	16.9	43	20.6
Developing or updating Nursing care plans/options	97	51.3	124	59.3
Educating patients and families	122	64.6	138	66.0
Proper documentation of Nursing care	103	54.5	115	55.0
Skin care	84	44.4	107	51.2
Comforting/talking with patients	130	68.8	142	67.9
Timely administration of prescribed medication	24	12.7	47	22.5
**Total**	**189**	**100**	**209**	**100**
